# Patient and Parent Well-Being and Satisfaction With Diabetes Care During a Comparative Trial of Mobile Self-Monitoring Blood Glucose Technology and Family-Centered Goal Setting 

**DOI:** 10.3389/fcdhc.2022.769116

**Published:** 2022-05-06

**Authors:** Jillian B. Halper, Lisa G. Yazel, Hala El Mikati, Amy Hatton, Jennifer Tully, Xiaochun Li, Aaron E. Carroll, Tamara S. Hannon

**Affiliations:** Department of Pediatrics, Indiana University School of Medicine, Indianapolis, IN, United States

**Keywords:** type 1 diabetes, patient-centered care, blood glucose monitoring, technology, psychosocial factors

## Abstract

Patient engagement in the process of developing a diabetes treatment plan is associated with person-centered care and improved treatment outcomes. The objective of the present study was to evaluate the self-reported patient and parent-centered satisfaction and well-being outcomes associated with the three treatment strategies utilized in a comparative effectiveness trial of technology-enhanced blood glucose monitoring and family-centered goal setting. We evaluated data from 97 adolescent-parent pairs at baseline and 6-months during the randomized intervention. Measures included: Problem Areas in Diabetes (PAID) child and parent scales, pediatric diabetes-related quality of life, sleep quality, and satisfaction with diabetes management. Inclusion criteria were 1) ages 12-18 years, 2) a T1D diagnosis for at least six months and 3) parent/caregiver participation. Longitudinal changes in survey responses were measured at 6 months from baseline. Differences between and within participant groups were evaluated using ANOVA. The average age of youth participants was 14.8 ± 1.6 years with half of the participants being female (49.5%). The predominant ethnicity/race was Non-Hispanic (89.9%) and white (85.9%). We found that youth perceived 1) greater of diabetes-related communication when using a meter capable of transmitting data electronically, 2) increased engagement with diabetes self-management when using family-centered goal setting, and 3) worse sleep quality when using both strategies together (technology-enhanced meter and family-centered goal setting). Throughout the study, scores for self-reported satisfaction with diabetes management were higher in youth than parents. This suggests that patients and parents have different goals and expectations regarding their diabetes care management and care delivery. Our data suggest that youth with diabetes value communication *via* technology and patient-centered goal setting. Strategies to align youth and parent expectations with the goal of improving satisfaction could be utilized as a strategy to improve partnerships in diabetes care management.

## Introduction

The vast majority of adolescents with type 1 diabetes (T1D) have glycosylated hemoglobin (HbA1c) values above the recommended range, indicating suboptimal glycemic control (1, 2). It is well-known that adequate frequency of self-monitoring of blood glucose (SMBG), appropriate insulin dosing, and parental involvement in type 1 diabetes management are important to achieve good glycemic control in adolescents (3, 4). Despite the increasing availability of advanced technologies to improve glycemic control, for adolescents, there are multiple technological and social barriers to adopting these technologies (5–7). Moreover, adolescents are often reluctant to accept recommendations for increasing SMBG or insulin doses when they feel that it increases their diabetes-related distress and burden (8).

Diabetes management is associated with substantial burdens, necessitating ongoing assessment and treatment of mental health and diabetes-related distress during routine diabetes visits (9). Poor glycemic control is often associated with lack of sufficient SMBG and insulin dosing when there is significant family conflict and increased diabetes-related distress (10). Previous studies indicate both general and diabetes-specific family conflict are associated with decreased diabetes self-care in adolescents and deteriorations in glycemic control (11, 12), while improved family communication is directly related to improved SMBG and better glycemic control (13). In addition, shared goals and teamwork between adolescents and parents can decrease diabetes-related family conflict and improve patient outcomes (14).

Shared decision-making is a model of patient-centered care that encourages individuals or families to be actively involved in the medical decisions that impact their health. It is recognized that a personal interest and investment in the diabetes treatment plan from the adolescent with diabetes is associated with engagement with diabetes self-management and improved metabolic control of T1D (9, 15, 16). We previously created a patient decision aid to utilize with decision coaching to facilitate adolescent patient and parent alignment with goals for diabetes self-management (family-centered goal setting) (17). We then performed an intervention that combined real-time sharing of SMBG data, electronic messaging, and a clinic-based family-centered goal setting strategy to address patient-centered diabetes care and family-centered goals simultaneously (18). The objective of previously published study was to compare 3 strategies for improving SMBG and diabetes outcomes in the short-term (6 months). These strategies were: (1) a technology-enhanced blood glucose meter that both shared blood glucose data among patients, their parent, and health care providers, allowing for text messaging; (2) family-centered goal setting; and (3) a combination of (1) and (2). Utilizing a family-centered goal setting strategy was associated with improved short-term outcomes when introducing new technology for SMBG. However, there is little known about shared decision making with adolescents with diabetes care and whether or not patients and families have an improved experience with healthcare teams who implement this into practice (19).

The objective of the present study was to evaluate the self-reported patient and parent-centered satisfaction and well-being outcomes associated with the three intervention strategies utilized in the published comparative effectiveness trial (18). We hypothesized that the combination of a technology-enhanced blood glucose meter along with family-centered goal setting would result in higher levels of patient-centered satisfaction with diabetes care.

## Materials and Methods

This study was performed at Indiana University Health, Indianapolis, IN, approved by the Indiana University Institutional Review Board, and all participants signed informed consent/assent prior to enrolling. Inclusion criteria were 1) ages 12-18 years, 2) a T1D diagnosis for at least six months and 3) parent/caregiver participation. Exclusion criteria included diagnoses of other chronic diseases except depression, asthma, and thyroid disease which, if present, were required to be controlled. We did not exclude these conditions because they are commonly present in youth with T1D and when controlled with stable doses of medications would not interfere with the intervention strategies. We excluded individuals currently using a continuous glucose monitoring system at the time of study entry because one of the intervention strategies included a technology-enhanced blood glucose meter and the duplicative systems could contribute to burn-out and/or not using the assigned intervention strategy. The complete methods and primary clinical outcomes of the parent study have been published elsewhere (18). In short, we identified potential participants from existing pediatric and adolescent diabetes clinics, pre-screened them for inclusion in this study, and invited eligible participants to enroll. After enrollment but prior to being randomized, participants entered a 3-month run-in period of routine diabetes care in an adolescent diabetes clinic to establish baseline characteristics and to accommodate for any enrollment effect on HbA1c prior to implementing the study intervention strategies. A single diabetes care provider (a board-certified pediatric endocrinologist) and a certified diabetes educator/nurse practitioner (CDE, CNP) provided American Diabetes Association endorsed care recommendations during the 3-month run-in period. Standard recommendations were made to perform SMBG at least 4 times per day (fasting, before meals, before bedtime), review SMBG records weekly, and dose insulin as prescribed prior to meals and snacks. Participants were then randomized using block randomization, stratifying by sex, in a 1:1:1 ratio to one of three treatment strategies: meter (n=43), goal setting (n=42), or meter/goal setting group (n=43). This study design allowed for the measurement of longitudinal change in outcomes in individuals by treatment, as patients were serving as their own controls, and between treatment groups.

The sample size estimation for the proposed pilot study was based on change in HbA_1c_ at 6 months from baseline (the mean of 2 measures taken at the onset and the end of run-in). For evaluation of the longitudinal change in outcomes measures in individuals by treatment (patients serve as their own controls): With 90 participants, we had over 90% power to detect a HbA_1c_ difference of 0.5 ± 0.8% from baseline to 6 months among individuals (paired-T tests, α = 0.05). For evaluation of treatment group differences for the 3 treatment arms: With 30 participants per group, we had 80% power to detect a HbA_1c_ difference of 0.5 ± 0.5% (one-way ANOVA, α = 0.05).

### Research Study Visits

After the run-in period, consented patients and parents were randomized to a treatment group during the baseline visit, therefore study activities were based on the allocated group, previously published (18). Each study visit consisted of meeting with the healthcare providers for routine diabetes care and physical examination, discussion related to the specific intervention strategy, and the completion of study questionnaires.

### Intervention Strategies

*HIT-enhanced SMBG strategy* - The Telcare System (Concord, MA, https://telcare.com) allowed for real-time SMBG data monitoring for the patient, their parents, and their healthcare provider by automatically transmitting SMBG data to a secure, online, HIPAA-compliant web portal *via* cellular data networks. Both pre-set and free-text alerts could be sent to any cell phone in real time when SMBG was performed. If randomized into this strategy, participants were encouraged to use the Telcare blood glucose meter throughout the study, regardless of their insulin delivery mode. Regardless if the patient used the intervention strategy meter or another meter, all data were downloaded and reviewed weekly by a CDE and a CNP. Adolescents were consequently either messaged through Telcare system with care recommendations or when possible, directly contacted by phone/text message.

*Family-centered goal setting strategy* – In addition to meeting with a health educator and a CDE, families randomized into this strategy met with a board-certified pediatric endocrinologist at the randomization visit. Using motivational interviewing, the multidisciplinary team helped the families set goals that were tailored to each family’s desires, while considering the age and stage of maturity based on that age of the participating adolescent. The goal-setting tool utilized was specifically designed for this study by a patient advisory group and is published in detail elsewhere (17). This tool was used to facilitate identification of diabetes self-management skills or behaviors that both the parent and adolescent agreed were important to work on and a reward system was discussed. Examples of self-management behaviors included specifics regarding self-monitoring of blood glucose, insulin dosing at meals, and adjusting insulin, but these were specific and unique for each parent-adolescent pair depending on their input. Independent from adolescents, parents chose goals that they felt were important and achievable for both themselves and their child. Conversely, adolescents chose goals that they felt were important and achievable for themselves and their parents. Tool responses were exchanged, and a shared decision-making process was utilized to choose mutually agreed-upon goals, tracking systems, and rewards.

For the present study, we focused on the impact of using this process on self-determined quality of life outcomes and diabetes distress.

As an example of a tracking and reward system, parents and adolescents were offered a jar and marbles in two different colors. Written on the jar were the agreed upon goals and rewards. Each time a behavior was demonstrated by either the parent or the adolescent, a marble of the corresponding color could be added to the jar. The adolescent could give the parent points and vice versa. Once an agreed upon number of marbles were added, an agreed-upon reward could be anticipated. This was not a required activity but was offered as an example to give a visual reminder of the goals that they had agreed upon.

Combined strategy – families participated activities for both the HIT-enhanced SMBG and family-center goal setting strategies. This required being instructed on the Telcare System and meeting with the research team to complete all the activities for the family-centered goal setting.

### Data Collection Instruments

Validated questionnaires were administered to youth and parent participants to evaluate patient-centered outcomes measures. All questionnaires were self-administered *via* a tablet, and data were stored in Research Electronic Data Capture system: REDCap (Vanderbilt University, Nashville, TN, USA), a HIPAA-secure database system approved by the university and healthcare system (20). The youth participants completed five questionnaires at baseline and six months: 1) The Problem Areas in Diabetes Validated Scale (PAID-Peds) measured burden related to T1D management and related emotional distress (21); 2) The Pediatric Quality of Life Inventory (PedsQL) version 3.2 was an exploratory measure to assess health-related quality of life and youth participants completed the appropriate PedsQL version for their age, 8-12 years or 13-18 years (22, 23); 3) The Cleveland Adolescent Sleepiness Questionnaire was collected to assess sleepiness and alertness given the emerging data of higher sleep disturbances in youth with type 1 diabetes (24); 4) The Adherence in Diabetes Questionnaire measured behaviors regarding T1D management and treatment (25); and 5) an original patient satisfaction questionnaire was utilized to assess satisfaction with diabetes management during the study at baseline and six months (**Supplementary Table 1**).

Participating parents completed three questionnaires at baseline and six months: 1) Problems Areas in Diabetes (PAID) measured emotional distress and burden with the adolescent’s diabetes management (26); 2) The Parental Environment Questionnaire Parent-Child Conflict Scale measured parent-child conflict (27); and 3) an original parent satisfaction questionnaire (**Supplementary Table 1**) was utilized to assess satisfaction with their adolescent’s diabetes management during the study. The original satisfaction questionnaires were scored so that a higher score reflects a higher satisfaction level.

### Analysis

We modeled the longitudinal changes in questionnaire responses at six months from baseline. In Statistical Package for Social Sciences version 26 (SPSS v26), we used repeated one-way ANOVA to compare the differences within and between subjects. Linear mixed effects models with random intercepts for subjects were used to model the longitudinal change in questionnaire responses at six months from baseline. The Kenward-Roger approximation was used to test within-subject differences between responses at baseline and 6 months for each intervention strategy group. A likelihood ratio test was used to measure this longitudinal change difference across the three groups. Data were assumed to be missing at random (MAR), and all analyses were conducted in R version 4.0.4 (2021-02-15). The linear mixed models were fit using the lme4 package.

## Results

One hundred twenty-eight participants enrolled, and 102 families completed the run-in period and were randomized to one of three intervention strategies. Ninety participants completed both the baseline and 6 months study visits. Baseline characteristics of the participants are shown in **Table 1**. The average age of youth participants was 14.8 ± 1.6 years with half of the participants being female (49.5%). The predominant ethnicity/race was Non-Hispanic (89.9%) and white (85.9%).

**Table 1:** Patient characteristics at baseline by intervention strategy assignment.

	Meter (N=35)	Goal Setting (N=34)	Meter/Goal Setting (N=30)	P-value
**Age (years)**				0.696^1^
Mean (SD)*	14.7 (1.8)	14.9 (1.5)	15.1 (1.6)	
**Female, n (%)**				0.991^2^
Female	17 (48.6%)	17 (50.0%)	15 (50.0%)	
Male	18 (51.4%)	17 (50.0%)	15 (50.0%)	
**Race, n (%)**				0.308^3^
White	32 (91.4%)	30 (88.2%)	23 (76.7%)	
Black	3 (8.6%)	2 (5.9%)	4 (13.3%)	
Other	0 (0.0%)	2 (5.9%)	3 (10.0%)	
**Ethnicity, n (%)**				0.433^3^
Hispanic	1 (2.9%)	1 (3.0%)	3 (11.1%)	
Non-Hispanic	33 (97.1%)	32 (97.0%)	24 (88.9%)	

*Mean age of the entire study group = 14.8 ± 1.6 years.

^1^Linear Model ANOVA.

^2^Pearson’s Chi-squared test.

^3^Fisher’s Exact Test for Count Data.

Baseline and six-month questionnaire responses are shown in **Table 2**. For child questionnaires, responses were comparable across the groups for all questionnaires at baseline. The scores for PAID-Peds did not change significantly from baseline to 6 months in any group and there were no between group differences in this measure during the study. The PedsQL scores for diabetes symptoms, treatment barriers, treatment adherence, and worry did not change significantly from baseline to 6 months in any group and there were no between group differences in these subscale measures. The PedsQL communication subscale score was increased at 6 months in the HIT-enhanced SMBG (meter-only) group (p=0.046). The total score on the Cleveland Adolescent Sleepiness Questionnaire changed only in the group assigned to combination therapy (p=0.022), indicating worse self-reported sleep quality, but there were not between group differences for change in this measure. Adherence in diabetes scores increased in youth participants assigned to the family-centered goal setting treatment strategy (p=0.01), but there were not between group differences for change in this measure. Satisfaction with the diabetes care plan increased in the HIT-enhanced SMBG (meter-only) group (p=0.047), but there were not between group differences for change in this measure. For parents responses were comparable across the groups for all questionnaires at baseline except for the Parental Environment questionnaire, which was lower in the combination therapy group at baseline (p=0.03). There were not between group differences for changes in any measure over time. Throughout the study, patient satisfaction with diabetes care was higher than parent satisfaction (baseline and 6-month differences, p<0.001).

**Table 2:** Change in study outcomes by intervention strategy assignment modeled by linear mixed effects models with random intercepts for individuals.

	Meter			Goal Setting			Meter/Goal Setting			Between Groups
	Baseline	6 Months	P-value^2^	Baseline	6 Months	P-value^2^	Baseline	6 Months	P-value^2^	P-value^1^
Child Questionnaires*											
**Problem Areas in Diabetes (PAID-Peds)**	15.3 ± 13.2 (34)	12.6 ± 13.2 (35)	0.220	21.1 ± 17.5 (33)	15.2 ± 16.3 (28)	0.054	14.2 ± 14.6 (30)	16.2 ± 15.7 (30)	0.365	0.105
**Pediatric Quality of Life (PedsQL)**										
Diabetes Symptoms	61.1 ± 14.9 (35)	63.6 ± 18.1 (34)	0.265	56 ± 12.7 (34)	59.1 ± 8.6 (27)	0.351	57.2 ± 14.7 (30)	54.3 ± 18.3 (30)	0.271	0.214
Treatment Barriers	81.9 ± 16 (35)	85.4 ± 15.6 (34)	0.161	79.4 ± 17.7 (34)	79.4 ± 16.8 (27)	0.767	78.3 ± 18 (30)	83.2 ± 14.6 (30)	0.090	0.322
Treatment Adherence	88.9 ± 11.6 (35)	87.9 ± 11.9 (34)	0.626	83.3 ± 17 (34)	84.9 ± 13 (27)	0.966	87.7 ± 13.6 (30)	83.8 ± 15.6 (30)	0.075	0.399
Worry	67.7 ± 22.6 (35)	65.3 ± 24.6 (34)	0.517	51.3 ± 25.1 (34)	58.5 ± 20.8 (27)	0.109	61.7 ± 21.9 (30)	60.6 ± 23.7 (30)	0.800	0.220
Communication	81.8 ± 17.4 (35)	87.5 ± 17.2 (34)	**0.046**	74.2 ± 22.3 (34)	81.7 ± 18.3 (27)	0.080	84.8 ± 21.1 (30)	87.1 ± 15 (30)	0.466	0.641
**Cleveland Adolescent Sleepiness Questionnaire**	33.1 ± 10.1 (35)	33.8 ± 8.9 (34)	0.664	39.7 ± 10.1 (34)	37.1 ± 10.5 (27)	0.561	36.3 ± 11.1 (30)	39.9 ± 10.9 (28)	**0.022**	0.106
**Adherence in Diabetes**	4.0 ± 0.6 (34)	4.1 ± 0.6 (34)	0.224	3.9 ± 0.5 (33)	4.2 ± 0.4 (27)	**0.002**	3.9 ± 0.6 (29)	4.0 ± 0.7 (30)	0.407	0.182
**Satisfaction**	45.3 ± 8.7 (28)	48.4 ± 5.7 (30)	**0.047**	45.9 ± 9.6 (18)	47.6 ± 5.3 (21)	0.514	50.2 ± 5.8 (17)	45.4 ± 9.1 (28)	0.072	**0.022**
Parent Questionnaires*											
**Problem Areas in Diabetes – Parent Scale**	31.6 ± 6.6 (35)	30.8 ± 6.2 (34)	0.435	30.0 ± 6.6 (34)	29.7 ± 5.5 (27)	0.897	31.6 ± 4.4 (30)	29.7 ± 5.6 (29)	0.051	0.406
**Environment**	24.7 ± 5.8 (35)	24.0 ± 6.7 (34)	0.715	26.2 ± 7.2 (34)	24.6 ± 7.4 (27)	0.378	22.9 ± 3.1 (30)	23.5 ± 3.9 (30)	0.461	0.546
**Satisfaction**	34.6 ± 6.5 (25)	33.0 ± 9.0 (31)	0.287	37.3 ± 3.5 (21)	36.9 ± 3.5 (23)	0.153	36.4 ± 3.8 (19)	33.6 (8.2) (27)	0.178	0.490

^1^ P-value associated with the test of H_0_: the change in the outcome from baseline to 6 months is the same across groups.

^2^ P-value associated with the test of H_0_: no change in the outcome from baseline to 6 months.

*Outcomes reported as mean ± SD (n).

P values for significant changes are bolded.

## Discussion

In this study, we evaluated patient-and parent-centered outcomes in participants in a comparative effectiveness trial of (1) a blood glucose meter that both shared blood glucose data among patients, their parent, and health care providers, and allowed for text-message communication (HIT-enhanced SMBG strategy); (2) a family-centered goal setting strategy; and (3) a combination of (1) and (2) (18). In youth participants, we found: 1) increased self-reported diabetes-related communication in the HIT-enhanced SMBG strategy group, 2) increased self-reported engagement with diabetes self-management as measured by The Adherence in Diabetes Questionnaire in the family-centered goal setting group, and, 3) worse self-reported sleep quality in the combination strategy group. Throughout the study, scores for self-reported satisfaction with diabetes management were higher in youth than parents. This suggests that patients and parents have different goals and expectations regarding their diabetes care management and care delivery.

Youth participants who were randomized into the HIT-enhanced SMBG strategy (meter only) perceived an improvement in diabetes-related communication during the 6-month treatment period. The lack of adequate communication between providers and patients with T1D is one of the major reported barriers for care (28). Some studies have suggested that greater communication with providers can ameliorate perceptions of barriers which can ultimately improve diabetes self-management and outcomes (29). However, despite the reported improvement in communication in this group, there were no significant objective changes in HbA1c in this group (**Supplementary Table 2**) (18). It may be that youth perceived communication was improved due to technological transfer of data to parents/healthcare providers, yet this did not transfer to actions that changed diabetes self-management behaviors.

Youth in the family-centered goal setting group reported increased engagement with diabetes self-management. Despite this perception, there were not significant improvements in either SMBG frequency or HbA1c as reported in our primary outcomes paper (**Supplementary Table 2**) (18). This discrepancy between increased perception of engagement with diabetes self-management and no change in HbA1c or frequency of SMBG could be related to the subjectivity of the scale or to the low test-retest reliability of the Adherence in Diabetes Questionnaire, which has not yet been established for this questionnaire (25). It could also be that youth who verbalize patient-centered goals during treatment self-report better engagement with diabetes self-management because the goals are better aligned with the patient’s reality rather than being assigned to them. Additional study is needed to further develop this patient-centered team approach so that youth-verbalized goals are highlighted and supported. Although those with continuous glucose monitors were excluded from our study, there is likely value in family-centered goal setting in this population and further study would be beneficial.

Although we did not document any significant changes in PAID-Peds questionnaires reported by youth, we noted that our youth scores were low (<40) (30). This reflects that the participants had low emotional distress and negative emotions, even at baseline. This might have been related to the 3-months run in period which can provide an added level of comfort to the participating families. It also suggests that families and youth who volunteer to participate in diabetes care studies may differ in important ways from the general population of youth with diabetes. Thus, the findings from this study may not be translatable to all populations of youth with diabetes.

Introducing diabetes technology can be associated with diabetes-related distress and sleep-related concerns (31). The greater the mental and physical burdens of treatment, the more likely they are to interfere with other aspects of health, including sleep. We documented a significant decline in self-reported sleep quality in youth participants in the combination therapy group. Sleep disturbances have been reported to be related to alarm fatigue and fear of hypoglycemia (31). Although we did not collect quantitative data on sleep outcomes, we speculate that night-time sleep disturbances could be related to the increased time associated with the use of the blood glucose meter that shared data among patients, their parent, and healthcare providers.

Strengths of our study include the randomized-controlled prospective design and 3-month run-in period. The run-in period was designed to blunt the positive effect that participation in a research study, on its own, may have on diabetes self-care and satisfaction with care. In addition, this trial was performed in a real-world clinic-based setting and questionnaires were completed at these appointment times. The limitations include that the study was underpowered for some of the analyses comparing group differences in the primary study. There were analysis limitations based on the questionnaires chosen for this study. The PAID-Peds and Adherence in Diabetes questionnaires are validated for age groups up to 17 years and our inclusion criteria included 18-year-olds. We did not collect all the psychological variables or quality of life data in the parents, which precluded evaluation of psychosocial similarities/differences in parents and their children. Satisfaction data reflected the overall experience with the clinic visit and could not be specifically attributed to the device or data sharing aspects of the study. The Adherence in Diabetes questionnaire was validated in a sample of Danish adolescents and may not have construct validity in US adolescents. There were clinical limitations as well. Due to clinic time and resource constraints, the needed family support may have been insufficient for establishing new care routines. There was no study-related clinical psychologist or social worker to provide ongoing support during the study. Finally, some adolescents may have been in the honeymoon period after diagnosis which may have affected outcomes.

In conclusion, our data do not support our hypothesis that the combination of a technology-enhanced blood glucose meter along with family-centered goal setting would result in higher levels of well-being or patient satisfaction. Our data suggest that recommendations to provide added support for patient-centered strategies in the pediatric/adolescent diabetes clinic may be needed. Youth with diabetes value communication *via* technology and perceive that communication is improved by technology. Strategies to ensure receipt of this data and interim management based on technology-driven communication should be pursued. Youth with diabetes value partnership and patient-centered goal setting, but parents may be frustrated when they do not get the results that they (parents) expect. Strategies to align youth and parent expectations with the goal of improving satisfaction could be utilized as a strategy to improve partnerships in diabetes care management. Consideration should be taken with regard to self-care strategies including sleep habits. Sleep quality may be decreased with increasing burden of diabetes care. Furthermore, next steps in this research will include implementing a shared decision-making clinical process in a virtual format in preparation for diabetes clinic visits.

## Data Availability Statement

The raw data supporting the conclusions of this article will be made available by the authors, without undue reservation.

## Ethics Statement

The studies involving human participants were reviewed and approved by Indiana University Institutional Review Board. Written informed consent to participate in this study was provided by the participants’ legal guardian/next of kin.

## Author Contributions

JH, LY, and TH: responsible for the majority of writing of manuscript. HM and XL: responsible for analysis. AH and JT: responsible for study activities and data management. AC and TH: responsible for study design. All authors contributed to the article and approved the submitted version.

## Funding

Funding for this work provided by: Indiana University; National Center for Advancing Translational Sciences; National Institutes of Health; Agency for Healthcare Research and Quality, Grant/Award number: 5R24HS022434-05.

## Conflict of Interest

The authors declare that the research was conducted in the absence of any commercial or financial relationships that could be construed as a potential conflict of interest.

## Publisher’s Note

All claims expressed in this article are solely those of the authors and do not necessarily represent those of their affiliated organizations, or those of the publisher, the editors and the reviewers. Any product that may be evaluated in this article, or claim that may be made by its manufacturer, is not guaranteed or endorsed by the publisher.

## Supplementary Material

Supplementary Table 1 Participant Satisfaction with Diabetes Care Plan. 1=strongly disagree; 2=disagree; 3=undecided; 4=agree; 5=strongly agree.

Supplementary Table 2 HbA1c and Frequency of SMBG by treatment at baseline and 6-mo time points*. *Data are means ± SD (n).
