# Supplementary material for: Patient and Parent Well-Being and Satisfaction With Diabetes Care During a Comparative Trial of Mobile Self-Monitoring Blood Glucose Technology and Family-Centered Goal Setting
Source: Front Clin Diabetes Healthc. 2022 May 6;3:769116. doi: 10.3389/fcdhc.2022.769116 (PMC10012089; doi:10.3389/fcdhc.2022.769116)
Supplement: Supplementary Table 1 — Participant Satisfaction with Diabetes Care Plan. 1=strongly disagree; 2=disagree; 3=undecided; 4=agree; 5=strongly agree. [file Table_1.docx]

Supplementary Table 1. Participant Satisfaction with Diabetes Care Plan

|  |  |
| --- | --- |
| 1. Goals discussed and selected during my/my child’s diabetes appointment were important to me (or to my child).  2. My opinions were considered during my/my child’s diabetes appointment.  3. I had a say in my/my child’s diabetes care plan.  4. I feel like a partner in developing my/my child’s diabetes care plan.  5. My questions were answered to my satisfaction during my/my child’s diabetes appointment.  6. I was treated with respect during my/my child’s diabetes appointment.  7. I felt welcomed during my/my child’s diabetes appointment.  8. I felt confident that I can do what is asked of me/my child at the diabetes appointment.  Additional questions for youth only:  1. I feel comfortable talking with friends about everything I need to do to care for my diabetes  2. I feel able to talk with adults other than my parents (teachers, coaches, etc.) about everything I need to do to care for my diabetes.  3. I have at least one adult other than my parents that I can talk to about my diabetes. |  |
|  |  |

1=strongly disagree; 2=disagree; 3=undecided; 4=agree; 5=strongly agree
