# Supplementary material for: Patient and Parent Well-Being and Satisfaction With Diabetes Care During a Comparative Trial of Mobile Self-Monitoring Blood Glucose Technology and Family-Centered Goal Setting
Source: Front Clin Diabetes Healthc. 2022 May 6;3:769116. doi: 10.3389/fcdhc.2022.769116 (PMC10012089; doi:10.3389/fcdhc.2022.769116)
Supplement: Supplementary Table 2 — HbA1c and Frequency of SMBG by treatment at baseline and 6-mo time points*. *Data are means ± SD (n). [file Table_2.docx]

Supplementary Table 2. HbA1c and Frequency of SMBG by treatment at baseline and 6-mo time points*

| **Treatment** | **HbA1c (%) Baseline** | **HbA1c (%) 6 mo** |
| --- | --- | --- |
| **HIT-enhanced SMBG** | 9.0 ± 1.6 (33) | 9.0 ± 2.0 (33) |
| **Family-centered goal setting** | 9.0 ± 1.9 (33) | 8.7 ± 1.8 (26) |
| **Combined approach** | 10.1 ± 2.1 (31) | 9.7 ± 1.9 (31) |
| **Treatment** | **Tests/day Baseline** | **Tests/day 6 mo** |
| **HIT-enhanced SMBG** | 3.6 ± 1.9 (29) | 2.9 ± 1.3 (29) |
| **Family-centered goal setting** | 4.0 ± 1.9 (31) | 4.1 ± 2.5 (21) |
| **Combined approach** | 3.2 ± 1.8 (28) | 2.7 ± 1.3 (29) |

*Data are means ± SD (n)
